# Quo Vadis, Nanothermite? A Review of Recent Progress

**DOI:** 10.3390/ma15093215

**Published:** 2022-04-29

**Authors:** Mateusz Polis, Agnieszka Stolarczyk, Karolina Glosz, Tomasz Jarosz

**Affiliations:** 1 Łukasiewicz Research Network—Institute of Industrial Organic Chemistry, Explosive Techniques Research Group, 42-693 Krupski Młyn, Poland; 2 Department of Physical Chemistry and Technology of Polymers, Silesian University of Technology, 44-100 Gliwice, Poland; agnieszka.stolarczyk@polsl.pl (A.S.); karolina.glosz@polsl.pl (K.G.)

**Keywords:** nanothermite, energetic materials, thermite, high-energy materials

## Abstract

One of the groups of pyrotechnic compositions is thermite compositions, so-called thermites, which consist of an oxidant, usually in the form of a metal oxide or salt, and a free metal, which is the fuel. A characteristic feature of termite combustion reactions, apart from their extremely high exothermicity, is that they proceed, for the most part, in liquid and solid phases. Nanothermites are compositions, which include at least one component whose particles size is on the order of nanometers. The properties of nanothermites, such as high linear burning velocities, high reaction heats, high sensitivity to stimuli, low ignition temperature, ability to create hybrid compositions with other high-energy materials allow for a wide range of applications. Among the applications of nanothermites, one should mention igniters, detonators, microdetonators, micromotors, detectors, elements of detonation chain or elements allowing self-destruction of systems (e.g., microchips). The aim of this work is to discuss the preparation methods, research methods, direction of the future development, eventual challenges or problems and to highlight the applications and emerging novel avenues of use of these compositions.

## 1. Introduction

Nanothermites are one of the most recently discovered types of energetic materials, initially being referred to as metastable interstitial composites [1]. The term “nanothermite” has been established by approx. 2005, by which time nanothermites were considered to be a modification of classical thermite formulations, in which the dimensions of phases formed by the components of the formulations were on the order of nanometers [2].

Since reducing the particle size of solids allows for better homogenization of compositions [3], the development of pyrotechnic compositions has led to the use of compositions based on nanometric components. Thanks to the improved homogenization and the extremely different nature of elements resulting from nanometric fragmentation, nanothermite compositions are characterized by lower burning induction times, higher burning velocities or huge reaction heats, several times higher than organic explosives [4]. It also enabled the use of elaboration techniques, so far unavailable or difficult for other high-energy materials, such as core-shell preparation [5], electrophoretic deposition [6] or atomic layer deposition [7]. It also opens new perspectives for designing explosive devices and allows us to use high-energy materials in disciplines where it was not possible so far.

However, the improvement of high-energy parameters in comparison to micrometric compositions is connected with the increase of costs, sensitivity to initiation stimuli [8], complexity of technological processes, and appearance of numerous new problems of both scientific and technical nature [9,10]. The use of nanomaterials necessitates resolving issues associated with the agglomeration, pyrophoricity, high reactivity and toxicity of both the constituents of and the nanothermite formulations themselves, as well as achieving an understanding of ignition and combustion mechanisms or aging resistance [11]. Nevertheless, the past few years have seen a progressive increase in the number of published works on the subject of nanothermites.

The original motivation for the development of nanothermites was to replace lead-based energetic materials in percussion primer mixes, due to their extreme toxicity and environmental harmfulness [1,12,13,14,15]. This effort is on-going and has since become a pressing concern, particularly in regards to the use of lead-based primary explosives. The ability of nanothermites and nanothermite-based formulations to produce a rapidly propagating combustion and shock front [16] qualifies these materials as prospective replacements of lead-based primary explosives [17]. Although use in lead-free initiation systems is perceived as the main application of nanothermites and derived energetic materials, it is not the only one, as recent works postulated many other areas of application suitable to these materials, such as gas generation, micropropulsion and molecular delivery [18].

The aim of this paper is to present the methods, techniques and trends, which allow the preparation of nanothermite compositions, to discuss the research techniques broadly in a way that makes it easier for other researchers to select a technique and find reference data, and indicate the present and future applications and development of devices using nanothermite compositions. The articles covered also familiarize the reader with current and future scientific and technical problems that must be overcome to realize the full potential of nanothermites. Among the reviewed works, we placed particular emphasis on the various methods of investigating nanothermites and critically discuss the use of those methods, along with their advantages, drawbacks and the most relevant issues faced by researchers. We have also highlighted emerging applications of nanothermites and the newest methods of fabricating these compositions.

## 2. Preparation of Nanothermites

The methods for producing nanothermites range from the straightforward, such as dry mixing, to the sophisticated, such as electrophoretic deposition. Most commonly, mixing of the nanothermite formulation components in liquids or their resonant acoustic mixing are employed. Both these and more elaborate fabrication methods, such as sol-gel and vapour deposition methods have been one of the subjects of recent review works on those materials [3,18]. Other methods, such as multi-layer deposition and 3D printings have also recently found application in the fabrication of nanothermited.

A method for producing core-shell nanothermites utilising Al as a fuel was patented [19]. The method consists of dissolving a metal salt in a mixed aqueous-organic solvent, followed by suspending Al nanoparticles (10–500 nm) in this solution. This suspension is then treated with a reducing agent, to precipitate the reduced metal ions on the surface of the Al particles. Once the reducing agent is depleted, the outer metal shell of the particles reacts with oxygen, yielding a core-shell structure, whose core is composed of Al and whose shell is composed of a metal oxide, acting respectively as the fuel and oxidising agent. This “wet” synthesis process is relatively straightforward and does not require the use of an inert atmosphere, as the invention recommends the use of reducing agents (hydrazine, NaBH_4_), which release inert gases upon decomposition, but have the drawback of significant toxicity.

An interesting deviation from the more commonly seen choices of materials is to use boron as the fuel and core in a core-shell thermite formulation [20]. Although boron finds some use in energetic materials, it is often viewed as a problematic fuel due to its high ignition point. In the patented work, the “activated boron powder”, resembling a nanothermite is produced by mixing boron powder, metal oxide nanoparticles and an active metal powder. This is followed by ball-milling and suspending the formulation in an organic solvent containing a silane-based coupling agent, in order to produce a composite. The composite is then dried and granulated, yielding 50–200 μm grains of the composite, composed of boron particles, whose surface is decorated with nanometric metal oxide and metal particles. Although the composite is reported to exhibit lower ignition temperatures by 150–300 K when compared with non-modified boron powder, the existence of both active metal oxide and metal on the surface of the particles may significantly sensitise the composite to accidental initiation, particularly by mechanical stimuli.

Another approach to producing core-shell type thermites is to utilise a suspension of micrometric aluminium powder in N,N-dimethylformamide (DMF), which is then acidified and in which PVDF is dissolved [21]. This is followed by adding sodium dodecyl sulfate, metal salt and a urea solution to the Al suspension and finally by heating the entire system to 280–380 ${}^{\circ }$C. The process yields micrometric aluminium particles, whose surface is covered with nanometric metal oxide, constituting a pseudo-nanothermite. Although the system is reported to exhibit a significant heat of combustion, no further details about the performance parameters or even sensitivity of the formulation to mechanical stimuli are given. Similarly, it is unclear how the evaporation of DMF, which is a harmful substance, from the reaction mixture during heating matches the environmental friendliness and use of non-toxic substances claimed for the proposed method.

Electrospraying of a precursor solution is a novel method for producing nanothermite formulations that allows supplementing these formulations with a variety of additives. Suspensions of CuO and Al nanoparticles in a solution of nitrocellulose can also be deposited by electrospraying them onto various substrates, such as semiconductor bridges [22,23]. In an earlier work, however, an iodine-doped nanothermite was produced using an acetone dispersion of CuO and Al nanoparticles that contained dissolved nitrocellulose, to which iodine was added. Ultrasonication followed by electrospraying of this suspension resulted in micrometric (5–20 μm) composite particles that, in the case of iodine-supplemented suspensions, were also decorated with iodine crystals. Other additives, such as energetic materials can also be used to modify the main nanothermite formulations, as seen by mixed electrospraying/electrospinning of CL-20 [24] or electrospraying both RDX and poly(vinylidenefluoride) [6] alongside a standard Al/CuO. The use of electrospray deposition also allows substituting components of nanothermite formulations, e.g., CuO can be replaced with copper sulfate, which exhibits better solubility while maintaining its oxidative character [17]. Although composite particles can indeed be produced via the electrospray method and their dimensions are easily discerned and measured via, e.g., scanning electron microscopy (SEM), the subject of the grain size distribution of the resultant particles is rarely touched upon in the relevant works. Another application of high electric fields for the fabrication of nanothermite-style materials is exemplified by the use of electrospinning. In the report, fluorinated polymers were employed as the fiber-forming matrix, in which Al nanoparticles were embedded [25]. Although the use of electrospinning is an interesting approach, not only do the produced nanofibers lack an oxidising agent, but the choice of fluorinated polymers in particular can be viewed as questionable for applications related to energetic materials, as the decomposition products of such polymers are rather hazardous, both to humans and to the environment.

A different approach for producing nanothermites utilises electrophoretic deposition [26]. Electrophoretic deposition relies on the fact that some nanoparticles are intrinsically electrically charged and, with the application of sufficient electric field, can be made to precipitate on an electrode. In the reported work, Al and Fe_2_O_3_ nanoparticles were employed, both apparently bearing an intrinsic positive electrical charge, but differing in regards to the electric field necessary for their precipitation. This difference was exploited to produce a nanothermite with a well-defined bilayer structure, whose constituent layers could be adjusted in terms of thickness by modifying the conditions of the deposition process. An analogous approach was employed to producing an Al/WO_3_ nanothermite from a suspension of Al and WO_3_ nanoparticles [27]. In this case, however, the applied electric field was not manipulated in any way and no evidence for the formation of an ordered (e.g., layered) structure being produced was presented.

Core-shell nanothermites do not necessarily have to take the form of particles, as core-shell structured nanowires have been reported to be grown on a copper foam via electrochemical anodisation [28]. In this case, the copper foam was first anodised in an alkaline environment to produce Cu(OH)_2_ nanowires on the surface of the foam. These nanowires were then calcined, in order to transform Cu(OH)_2_ into CuO, followed by the deposition of Al onto the CuO nanowires via magnetron sputtering. Although the copper foam appears to be uniformly covered with the CuO/Al nanowires and their structural features will likely be easily tuned by altering the parameters of anodisation and magnetron sputtering, the fabrication process appears to be both labor- and cost-intensive, requiring fairly sophisticated instruments, which may hinder any but the most specialised applications of this material.

The use of high electrical fields can be effectively coupled with vapour deposition methods, as sequential crystal templating and magnetron sputtering was employed for producing an uncommon Al/NiFe_2_O_4_ nanothermite formulation [29]. The crystal templating step was conducted by in situ depositing NiFe_2_O_4_, from a solution of nickel(II) and iron(III) nitrates, onto a crystalline polystyrene template, which was removed via pyrolysis following the precipitation of NiFe_2_O_4_. Subsequently, Al was deposied onto the NiFe_2_O_4_ scaffold via magnetron sputtering. Although this approach allows an interesting, porous structure to be produced and “loaded” with Al, it requires multiple labour- and time-intensive steps that are unlikely to be easily scaled up. These limitations are expected to adversely affect the possibility of applying this nanothermite farbication method on a wider scale. Magnetron sputtering has also been used to produce multilayered Al/CuO nanothermites [30]; however, the need to cool the substrate for 600 s before the deposition of each layer effectively excludes any practical application of this method.

Although the sophisticated methods of fabricating nanothermite formulations can offer extensive process control or the ability to manipulate the components of the formulation, the more straightforward methods, such as ultrasound-assisted dispersion and mixing are are not without advantages, such as involving a lesser number of unit operations, being less time- and labour-intensive or not requiring access to expensive apparata. In the reported case, a fairly standard Al/Fe_2_O_3_ nanothermite formulation was produced by first dispersing Al and Fe_2_O_3_ in n-hexane under an inert atmosphere [31]. Mechanical mixing was also claimed to be used in an invention, but no relevant [32].

## 3. Characterization Techniques

Unusual properties of nanothermites, imply the application to their investigation, apart from typical techniques of investigating high-energy materials, also techniques developed with this group of compounds in mind. The aim of this chapter is to describe and characterize the most important research methods that allow to characterize nanothermite and NSTEX compositions as fully as possible. The idea of the chapter is to list research techniques suitable for this group of high-energy materials, which may facilitate the choice of research techniques.

### 3.1. Sensitivity

#### 3.1.1. Sensitivity to Mechanical Stimuli

The sensitivity of a high-energy material is most commonly referred to as the minimum amount of energy required to rapidly initiate an explosive reaction [33]. The friction sensitivity test is usually realized using Peters apparatus, according to the standards [34,35]. The idea of the test, consists in placing the test sample between a ceramic plate and a ceramic punch, attached to an arm whose design allows the suspension of weights. By changing the weight of the weights and their position, the pressure of the punch on the specimen expressed in N is adjusted. Actuation of the apparatus causes movement of the plate with the material under test and its seizure by the punch. The test is usually carried out until the upper explosive limit is determined (6 ignitions per 6 repetitions). Alternative techniques for determining friction sensitivity include the mallet friction test [36], sliding-block friction test [37], emery-paper friction test [36] and pendulum friction test [36].

The impact sensitivity is determined using different types of drop hammers. The most commonly used is the free-fall hammer BAM (Kast hammer), performing the determination according to the standards [38,39]. In this test, a sample of high-energy material is placed between two steel cylinders bounded by a steel sleeve. A hammer of a specified mass is dropped on such a set after being placed in the anvil. The rules of conducting the measurement are described in the standard [38]. The authors of [40] presented a series of objections to this method. However, other types of apparatus are also in use, e.g., according to Bureau of Explosives, Bureau of Mines (ERL), Picantinny Arsenal, ball-drop Rotter, Olin-Mathieson [41] or 30 kg free-fall hammer [37,42,43]. The technique using ball-drop apparathus is worth describing. In the case of this technique, a ½ inch diameter steel ball is dropped from a specified height (with a step of 1 inch) onto the sample layer of high-energy material. The advantage of this method, is the use of a sphere as the striking element (beater), which causes simultaneous impact stimulus on the sample and its compaction and obliteration [37,41].

There are testing techniques that allow a mixed mechanical stimulus to be applied to a sample. It should be noted that these techniques better reflect the actual sensitivity to mechanical stimuli than the techniques described above. This is due to the impossibility of such a simplified interaction of the actual initiating stimulus, under non-laboratory conditions. Among these techniques we should mention the so-called Torpedo Test. The idea of the measurement is to drop on the test sample, a torpedo-shaped tup at an angle of 70–80 degrees. Such tests as Susan test [36] in which one pound of explosive in a special projectile is driven against a hard target at selected velocities. It is really a form of impact test and is used only with secondary explosives [44,45]. The projectile is designed to simulate the situation of collapse, in a manner of squeeze and nip on explosive between metal surfaces in the process. Projectile is accelerated by an air-gun or propellant-gun to hit a massive steel target with a certain velocity. The flying velocity of the projectile is measured by a timekeeping system to evaluate the sensitivity of the explosive charge. In addition, similar tests requiring a large explosives mass such as the Steven [45,46,47] test or the spigot [46] test are not applicable to the study of nanothermites. Some of these techniques found application among others in works [48,49,50,51,52].

#### 3.1.2. Sensitivity to Electromagnetic Radiation

The mechanism of high-energy materials initiation by radiation is related to thermal, resonance, and photochemical effects [53]. Currently, there are no standards describing the methodology of testing sensitivity of high-energy materials to laser radiation, despite many works on this subject [54,55]. The key parameters describing the high-energy materials sensitivity to laser radiation are the radiation density (expressed in J/cm^2^) and the ignition induction time for a given laser power and radiation length. The paper [56] describes the simplest possible system where the laser radiation, after passing through a collimator, is directed straight at the high-energy material sample, placed inside the measurement chamber. The signal triggering the laser is synchronized with the photodetector. In such a measuring system, the radiation density must be determined independently for given initial conditions. The control and data recording system includes a digital oscilloscope, which simultaneously records the time of the radiation effect and the signal coming from the photodetector.

**Figure 1:**
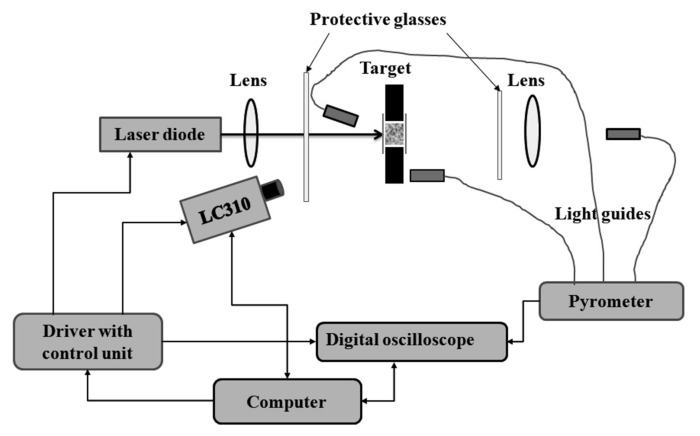
Research stand for determination of laser radiation sensitivity of explosives. Reprinted from [58] with permission form Elsevier.

Similar research stand was described in work [55]. The paper [57] describes a test system in which a beam splitter is additionally used before the radiation falls on the sample, to record pulse duration and energy (radiation) density. In the system presented in paper [58], showed in Figure 1. an important modernization has been applied. In this system, the radiation generated by the laser, after passing through the optical focusing system is directed to the sample. The power of the radiation, however, is determined using an optical power meter placed behind the sample. The response of the sample is determined by means of a set of pyrometers and a high-speed frame camera. The coupling used allows the laser to be turned off automatically when ignition is initiated.

The critical energy density required to initiate reaction was determined by numerical integration of the dependence of radiation intensity versus time.

A different solution to the power measurement issue was described in work [59]. An optical power meter was placed in the front of the sample. In the work [60], a complex system was used to allow simultaneous testing of laser sensitivity and pressure parameters. The design of the apparatus allows for testing in different atmospheres and for different initial pressures. Interesting research setups are also presented in the papers [61,62]. The nanotermites such as Al/CuO was examined for radiation sensitivity in works [28,58,63,64,65], Al/NiO [66], Al/Mn_2_O_3_ [67], Al/TiO_2_ [65], Al/MnO_2_ [65], Al/CoFe_2_O_4_ [5], Al/CuFe_2_O_4_/GO [61], Al/PVDF [68], Al/KClO_4_/GO/NC [62], Al/NiFe_2_O_4_ [29], Al/Fe_2_O_3_ [69,70,71], Al/Bi_2_O_3_ [58,63,64], Al/MoO_3_ [58,63], NC/Al/RDX [72] and Al based nanothermites with fullerens [73].

In work [74], the authors examined the Al/MoO_3_ nanothermite, by using setup similar to described in work [59]; however, an unusual detection method was used. The authors point out that thermites reactions are characterized by strong optical emission, which can make imaging with a standard high-speed camera difficult. At the same time, the use of neutral density filters limits the quality of the obtained image. The authors described a system using a coupled high-speed camera with a pulsed copper vapor (CV) laser. In this system, the camera captures the reflected laser light from the sample and the moving reaction front.

**Figure 2:**
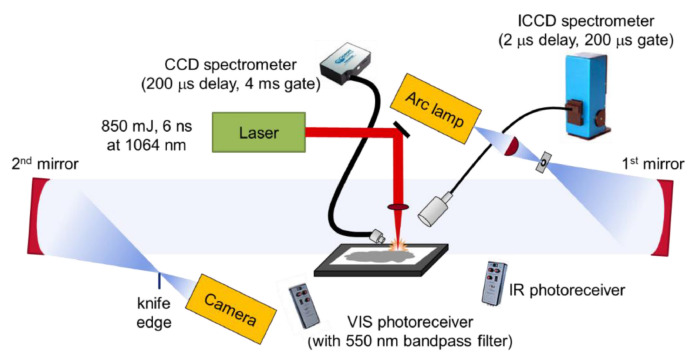
LASEM experimental setup with schlieren imaging and emission diagnostics. Reprinted from [75] with permission form Elsevier.

Interesting study was presented in work [75]. The authors examine the binary and ternary composition of Al, CuO and Bi_2_O_3_ with LASEM (laser-induced air shock from energetic materials) [76] technique (Figure 2). The technique is based on the generation of nanosecond laser pulses that initiate the reaction of high-energy material in conditions similar to those existing during high-energetic processes. The impact of the laser pulse of high energy and small duration leads to the generation of a plasma layer from the ablated material, with a sufficiently long duration of the radiation impact there is a plasma shielding phenomenon [77] leading to an increase in plasma temperature. After the end of the laser radiation exposure, the limited cooling of the plasma leads to a rapid expansion of the ionized compressed products, which interacting on the piston principle generate shock waves in the surrounding air [78]. When interacting with high-energy materials using this technique, the structure and parameters of the generated shock wave are strongly moderated, depending on the characteristics of the explosive under study. In the mentioned work, the authors determined the characteristic velocity of the laser-initiated shock wave, the emission intensity and the amount of total heat released.

The used system, based on [78] is presented above.The sample was placed on a vertical table in the test section between two mirrors, the laser pulse was focused on the sample surface using a lens. To visualize the laser-induced shock wave generated in air, the authors used the schlieren [79] imaging technique. In the system discussed here, an arc lamp served as the source of illumination, which was focused on the first mirror using an aspherical condenser lens. The light was collimated between the two schlieren mirrors. Changes in the refractive index of air due to both exothermic reactions and shock wave motion affect the trajectory of the light from the arc lamp, allowing imaging of both the heated air and the shock wave. A high-speed color camera was used for recording. To determine the position of the shock wave, the authors of this paper used a proprietary program created in Matlab. The described system was also equipped with a CCD spectrometer, an echelle spectrometer and an ICCD. The system was optimized so that the registration of the CCD spectrometer starts with a certain delay, in order to avoid the registration of the emission generated by the plasma and to register only the emission of the burning particles. Accordingly, a photoreceiver sensitive in the infrared and in the visible light range equipped with a bandpass filter was used to record the emission over time by the plasma and the BO_2_ emission over time.

Except for the techniques mentioned above, the sensitivity for microwave radiation needs a separated mention. The paper [80] presents a model of thermite ignition by microwave. Research on this issue has been discussed in [81] for Al/Fe_2_O_3_/GO/rGO compositions and [82] where Al/Fe_2_O_3_ thermite was studied. The example research constructions, were described in works [80,82,83,84,85].

#### 3.1.3. Sensitivity to Electric Discharge

In the document [41], there are four methods listed for testing explosive sensitivity to electric spark, successively, NSWC (method 1031), ARDEC (1031), NAWC (1033) and large scale test (1034). Moreover, this issue in the context of rocket propellants is described in the European standard [86]. The idea of each of these methods is based on generating an electric spark between electrodes separated by the sample of the high-energy material. Usually, the spark generation system is based on capacitors, which is related to their ability to rapidly release charge. Knowing the capacitance and voltage of the capacitors used, the energy of the discharge can be determined according to the formula [56]:

(1) $$E=\frac{CU^{2}}{2}$$

Usually, the measured parameters include minimal electrostatic energy necessary for ignition, maximum electrostatic energy where no ignition occurred and electrostatic energy with 50% probability of explosion [87,88]. According to [41,89], the measurement consists of discharging the system under test for assumed initial conditions. If no ignition occurs, the measurement is repeated until 20 repetitions are obtained (non-go). If explosive is initiated, the spark energy is lowered until no ignition is obtained in 20 trials. The differences between the methods relate to the shape of the electrodes, the weight of the high-energy material under test, the distance of the electrode from the sample, the method of initiating the measurement, the capacitor set used and the test methodology. For example, studies in accordance with aforementioned standards, are presented for Al/In_2_O_3_ [50], Al/SnO_2_ [49], Al/MnO_2_/CNF [52], Al/SnO_2_/polyaniline [90] or Al/SnO_2_/polypyrrole [91].

**Figure 3:**
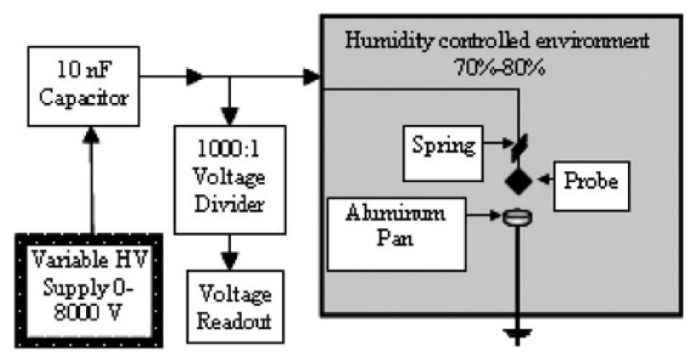
Schematic of ESD sensitivity tester. Reprinted from [93] with permission of John Wiley and Sons.

In [92], the study of static electricity susceptibility (of Al/CuO nanothermite) was performed with a different method than the ones described above. The bulk density high-energy material under test was placed in a 4.5 mm diameter, high polymer tube placed between the electrodes. The capacitors were then discharged in a given arrangement and the results were recorded. The authors of this paper took as the minimum ignition energy the value between no ignition in 20 repetitions and ignition in each of 20 trials. Due to the extremely high sensitivity of nanothermites to electrical sparks, the measurement range of typical instruments may not be sufficient. In [93], a system dedicated to the study of nanothermites is described (Figure 3), allowing the generation of a very low energy spark. Samples were placed in aluminium pans, with a volume of 0.05 cm^3^. An electrode was then manually applied to the test sample to discharge the capacitor. If no ignition occurred, the test was repeated by increasing the discharge energies. The minimum energy for reliable ignition was considered to be the discharge energy for which six ignitions occurred out of six trials. The insensitivity threshold was determined by decreasing the discharge energy from the minimum ignition energy until there was no response for 6 trials. The measurement system used is shown in the figure below.

In work [94], nanothermites based on Al and CuO, Fe_2_O_3_, MoO_3_ and those nanothermites with addition of Viton or Palmitic acid as a desensitizing agents were studied. Analogous to the work [93], the limits of sensitivity and insensitivity were determined based on six equal tests, by using an analogous arrangement as in [95].

The human body model (HBM)-based apparatus, described in [95], simulates a charge jump from a human body. In this system, a specimen placed in a nylon body is subjected to a load by a spark after the top insulating layer (tape) is pierced by a pin electrode. The tests were performed using the brucetown (stepped) method [96]. The same apparathus and conditions was applied for examination of Al/MoO_3_ nanothermite in work [97]. In addition to those described above, the issue of testing the sensitivity of nanothermites to ESD was discussed for Al/Ta_2_O_5_ and Al/Nb_2_O_5_ [98], Al/CuO [88], Al/Bi_2_O_3_/NC [87], Al/WO_3_/CB [99], Si/NaClO_4_ [100], Al/CuSO_4_·5H_2_O and for Al-based nanothermites with different sulfate-salt nanothermites [101].

#### 3.1.4. Sensitivity to Thermal Stimuli

To study the properties of high-energy materials (including nanothermite) as a function of temperature, DTA, DSC and TG techniques are most commonly used. These techniques allow observing whether exo- or endothermic transformations take place and determining their parameters, including reaction rate constants, activation energies, mass loss, etc. They are frequently applied for studying the stability and compatibility of energetic formulations, as well as for flash point investigations. They are an important element that allows a comprehensive analysis of the combustion process, determining the limiting stages and the complexity of the mechanism. These techniques are commonly used and little could be said about the methodology of their use in investigating nanothermite formulations that could not be said about their application in studying other types of energetic materials. One general remark that should be made, however, is that the gaseous environment employed in such studies has a significant effect on their results, with the primary distinction being between environments containing oxygen (e.g., synthetic air) and lacking it (e.g., nitrogen). To exemplify, ignition temperatures observed for measurements conducted in inert gases (e.g., argon), are commonly lower than those observed during measurements conducted in air, due to the occurrence of reactions between the fuel and oxygen. In some cases, the nitrogen atmosphere can affect the total combustion heat, due to possibility of reaction between sample and the gas. Due to the fact that most nanothermite formulations are in the form of readily flowing powders, the contact between the sample and sample holder can be considered as fairly high and is not expected to cause significant issues for measurements, particularly if low temperature scanning rates are employed. However, it is necessary to mention the studies aimed at determining the ignition temperature, under conditions close to the real conditions occurring at the combustion front. As is well known, with increasing heating rate, the recorded ignition temperature of high-energy materials also increases [102]. This is related to the delayed heat transfer to the material under examination and its dispersion throughout the material. Typical heating rate values for these techniques are in the range of 1–40 K/min, when heating rates at the burning front reach 10^6^ K/s [10,103], and in the case of shock initiation even 10^8^ K/s [104]. One should also pay attention to the fact that usually a few milligram weights are tested, which has a significant influence on the result. Even instruments dedicated to explosives allow examination of a maximum of 50–250 mg. In spite of these drawbacks, these techniques have been applied in many works.

**Figure 4:**
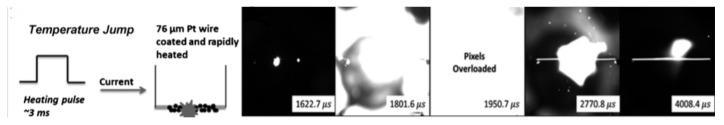
Schematic of T-Jump test idea and sequence of photos captured during the test. Reprinted from [105] with permission of John Wiley and Sons.

A particularly interesting technique to determine the ignition temperature is the T-jump technique. The idea of the test is to place a small path of high-energy material on the surface of a platinum wire or pedestal. The next step is to heat the resistive element to a temperature of the order of 1000–2000 [K] within a maximum of a few ms, with a current pulse of tens of amperes [23] (Figure 4).

The use of such a huge temperature rise rate (≅4−6 × 10^6^ K/s) eliminates the influence of the heating rate on the ignition temperature of the composition and allows neglecting the heat loss to the environment. Knowing the current parameters used in the study, the induction time, the dependence of resistance of the conductor as a function of time, the parameters describing the thermal and electrical properties of the conductor and recording the process with a high-speed camera [106] or a photodetector [107,108], it is possible to determine the flash point of the composition. When using platinum resistance elements, it is possible to use the well-known Callender–Van Dusen equation [109]. The T-Jump technique is often paired with mass spectrometry, the topic of which is discussed in more detail in Section 3.2.2. This technique, also allows us to calculate activation energy [110]. Usually, in research, authors used platinum wire, but in works [111,112], the authors used Ni-Cr wires, which are characterized with much more resistance than Pt wires, which allows us to apply lower currents. This technique is applied for examine such nanothermites as Al/CuO [23,113], Al/I_2_O_5_ [114], Al/Fe_2_O_3_ [104], Al/KClO_4_ [108], Al/NaClO_4_ [108], Al/AgO_3_ [115], Ti/KClO_4_ [116], Al/I_2_O_5_ [117], Si_2_O_5_ [117], Ti_2_O_5_ [117], Al/Bi_2_O_3_ [111], Al/Bi_2_O_3_/KClO_4_ [111], Al/MoO_3_ [112], Al and Ta with δ-HIO_3_, treatment of commercial HIO_3_ at approx. 180 ${}^{\circ }$C-HIO_3_, aerosol spray pyrolysis-HI_3_O_8_, treatment of commercial HIO_3_ at approx. 180 ${}^{\circ }$C-HI_3_O_8_, aerosol spray pyrolysis-I_2_O_5_, commercial-I_2_O_5_ [118], Al with persulfate salts [106].

**Figure 5:**
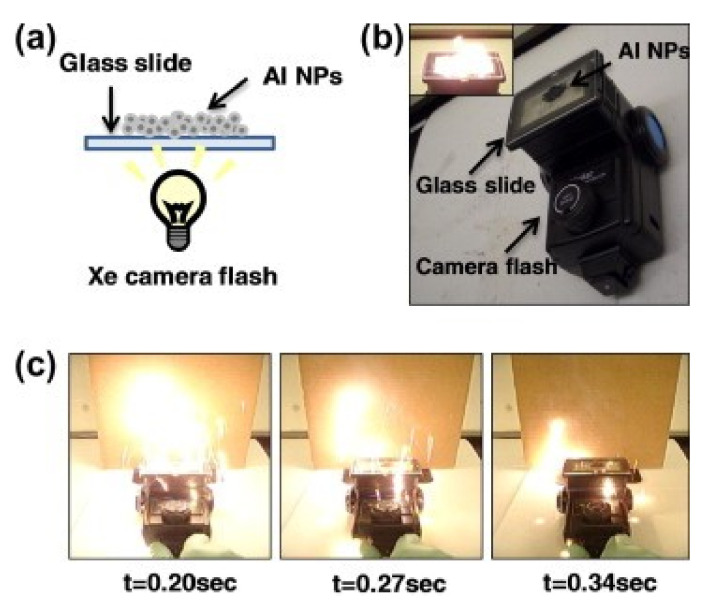
Flash ignition test idea and sequence of photos captured during the test: (**a**) Schematic; (**b**) optical images of the experimental setup; (**c**) Photographs of the combustion of an Al/CuO thermite. Reprinted from [121] with permission of Elsevier.

Different method used to measure a temperature of explosion in conditions similar to explosion regimes, is the flash ignition test. In [119] the authors investigated Al/WO_3_ nanothermite ignition using an optical flash ignition method based on the works [120,121], in the system described in [121] (Figure 5).

**Figure 6:**
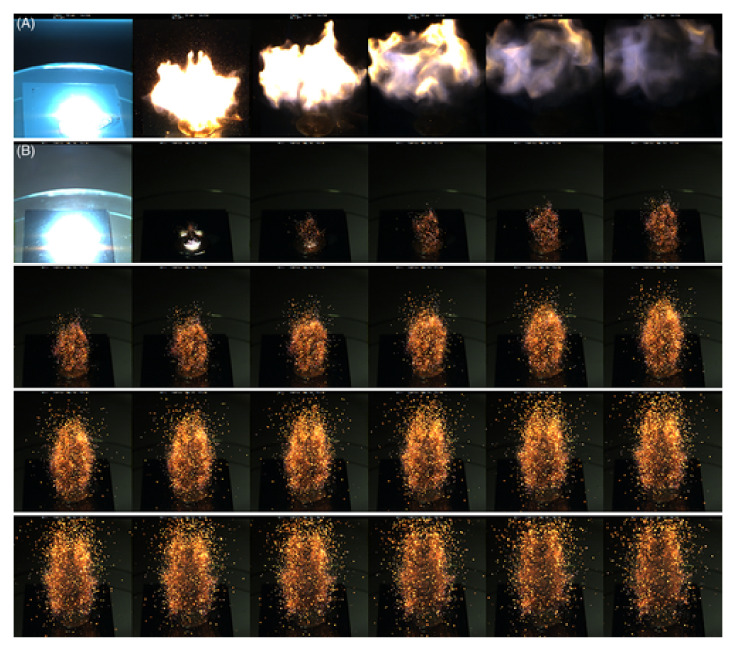
Sequence of photos captured during the flash ignition test. Photographs taken in 1 ms intervals, depicting the combustion of (**A**) Al/SnO_2_–PAni-7.7 and (**B**) Al/SnO_2_ thermites. Reprinted from [49] with permission of John Wiley and Sons.

The idea of the test is based on placing a test sample of high-energy material on a strictly specified transparent surface. Then a source of radiation (usually xenon lamp) with a specified energy density is placed below at a given distance. By recording the process of ignition of the composition it is possible to determine the ignition temperature and the energy required to cause it. The heating rate of the sample in this method reaches ∼10^5^ K [121]. This method was used to study, among others, Al/WO_3_ [122], Al/I_2_O_3_ [50], Al/SnO_2_ [49]. In the paper [123] Al/Fe_2_O_3_ nanothermite was studied using a similar measurement system (Figure 6).

In this setup, the authors placed 5 mg of the explosive on a 6×6 mm glass pedestal. The plate was then placed inside a xenon flash lamp, with an inner diameter of 15 mm. The large size disparity allowed the assumption that all of the radiation incident on the sample was uniform. By generating increasingly strong light pulses, the authors determined the minimum energy density required for ignition and temperature of explosion. Note the significantly lower heating rates—on the order of 10^3^–10^6^ K/s.

### 3.2. Chemistry and Morphology

#### 3.2.1. Heat of Explosion

The heat of thermite reactions can be determined by computational methods using thermochemical codes (such as TIGER, CHEETAH, EXPLO5 or CEA [115,124,125] or more complex computational models [126,127]. The indicated thermochemical codes are typically based on the prominent equations of state for explosives, such as the Becker–Kistiakowski–Wilson (BKW) equation in the case of TIGER and CHEETAH codes [128], or the Jones–Wilkins–Lee (JWL) as in the case of the EXPLO5 code [129] equations. Thermochemical codes can vary significantly in terms of assumptions about the reacting system or the relevant boundary conditions, i.e., EXPLO5 is limited to modelling combustion in either adaiabatic or isochoric conditions. It should also be noted that these methods allow only to a limited extent to take into account the heat loss and the non-ideal, real reaction regime. This parameter can also be determined on the basis of DSC or DTA studies, however, with all the limitations of this technique mentioned above. Depending on the chosen method, the measured or calculated values may be extremely different [130]. A typical method for determining the heat of reaction, is to use calorimetric bombs. Calorimetric bomb makes it possible to determine the total heat of reaction, making it possible in the case of detonation calorimeters, to study even several tens of kilogram samples of high-energy materials [131]. An important advantage is also the conduct of measurements in different atmospheres and pressures.

**Figure 7:**
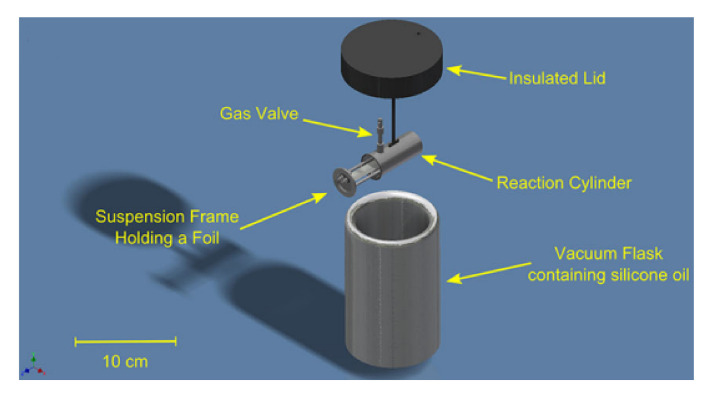
Expanded view of the calorimeter assembly. Reprinted from [137] with permission of Springer Nature.

The limitation of the majority of typical calorimeters in investigations of high-energy materials is their robustness, implying the use of small masses of investigated high-energy materials of the order of several tens—several hundred mg [132], when typically commercially available calorimeters are designed and optimized for testing of gram masses and for measurements at the level of several kJ of heat [133]. However, device design is required to maximize accuracy, precision, and sensitivity. In the case of testing such small masses, it is not possible to ensure appropriate, reliable measurement conditions. One of possible solutions is the use of microcalorimeters-miniaturized versions of calorimetric bombs, designed for milligram weights of samples [134,135,136]. When studying high-energy processes, extremely high sensitivity and low inertness of the devices are required due to their rapid nature. A description of such a device (Figure 7), is presented in [137]. The calorimetric bomb described allows for testing samples of 5–200 mg. The device in question consists of a titanium cylinder containing the substrate on which the sample is placed, the electrode lead and a gas valve. This cylinder is immersed in a vacuum body filled with silicone oil. The use of a titanium body with a lower heat capacity than steel and silicone oil in place of water (2.62 times lower specific heat) maximizes the sensitivity of the device and reduces its inertia. The designs described in the above mentioned papers, vary in the mounting of the sample and the ignition system.

Calorimetric techniques found application among others in examination of nanothermites such as: Al/KClO_4_ doped with GO/rGO/CNT/CNF [138], Al/B/Fe_2_O_3_ [132], Ta/WO_3_ [139], Al/CuO [140], Si/NaClO_4_ [141] or Al/I_2_O_5_, Al/AgIO_3_, Al/Ca(IO_3_)_2_, Al/KClO_4_ [130].

#### 3.2.2. Characterisation of Morphology and Composition

Nanothermite and NSTEX-type compositions show high sensitivity to the quality and parameters of the components used in their production. It is particularly important to pay attention to the fragmentation, even a small disruption of this parameter implies rapid changes in high-energy properties [3]. In addition, preparation processes such as electrosputtering, electrophoresis or deposition of atomic layers in a magnetic field require rigorous quality control of the obtained systems. An important element of research allowing postulating about the reaction mechanism is the study of the composition of reaction products, their composition, morphology, crystallographic form also in real time—during the high-energy transition. The techniques described in the following chapter are applicable both in the evaluation of raw materials, preparation and serve as a source of data to determine the nature and mechanism of reactions occurring during high-energy processes. As the realization of investigations for the discussed group of high-energy materials does not imply any changes in the design of the apparatus or the method of measurement, it is not advisable to discuss in detail these well described in the literature and known techniques within the framework of this work.

The microscopic techniques most commonly used include; scanning electron microscopy (SEM) [11,62,91,111,127,142,143,144,145,146,147,148,149,150,151,152,153], transmission electron microscopy (TEM) [5,92,114,119,138,142,146,154,155,156,157,158,159,160,161], atomic force microscopy (AFM) [142,153] and field emission scanning electron microscopy (FESEM) [112,143,156,162,163]. These techniques differ in imaging principle, resolution, and possible applications. It is important to note the high popularity of the SEM technique due to its relatively low cost and sufficient resolving power for most applications. SEM microscopes are often equipped with EDS (energy dispersive spectroscopy) detector enabling simultaneous, rough qualitative and quantitative identification of the studied sample. Techniques to identify the chemical composition include mass spectrometry [106,108,115,116,164,165,166,167,168,169,170], X-ray diffraction (XRD) [5,11,31,91,107,114,138,143,171,172,173,174,175,176,177,178,179,180,181,182], fourier-transform infrared spectroscopy (FTIR) [72,138,143,149,150,151,160,175,180,183,184,185,186], Raman spectrometry [61,81,100,138,143,179], Nuclear magnetic resonance spectroscopy (NMR) [73], X-ray photoelectron spectroscopy (XPS) [8,81,107,162,187].

A fascinating modernization of the TEM technique was presented by the authors of the paper [188]. By using a special designed probe, they performed an in situ study involving heating of nano-Al at a rate of 5 × 10^5^ K/s to a temperature of 1473 K directly in the TEM microscope chamber. In [189], the authors performed an analogous study for nano-Al particles and Al/WO_3_ compositions by pairing a heating probe with an SEM microscope and achieving heating rates on the order of 10^6^ K/s.

**Figure 8:**
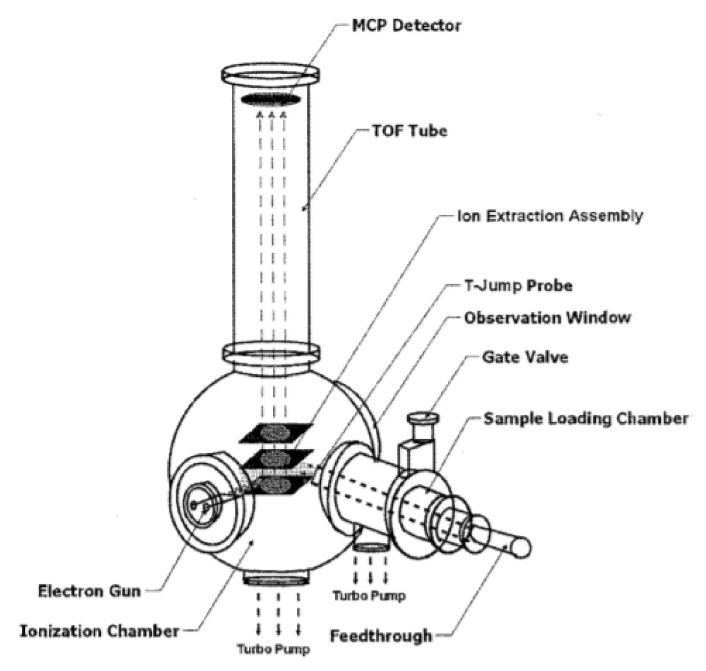
T-Jump/TOFMS probe. Reprinted with permission from [164]. Copyright 2010 American Chemical Society.

However, the T-jump technique combined with TOFMS requires a detailed description. Ions are accelerated by an electric field of known intensity. This acceleration causes the ion to have the same kinetic energy as any other ion of the same charge. The ion’s velocity depends on the ratio of its mass to its charge (heavier ions with the same charge achieve lower velocities, although ions with higher charges will also increase their velocity). The time it takes for the ion to reach a detector at a known distance is then measured. This time will depend on the velocity of the ion, and is therefore a measure of its mass-to-charge ratio. In works [164,165,190,191], the authors proposed a new research setup (Figure 8).

**Figure 9:**
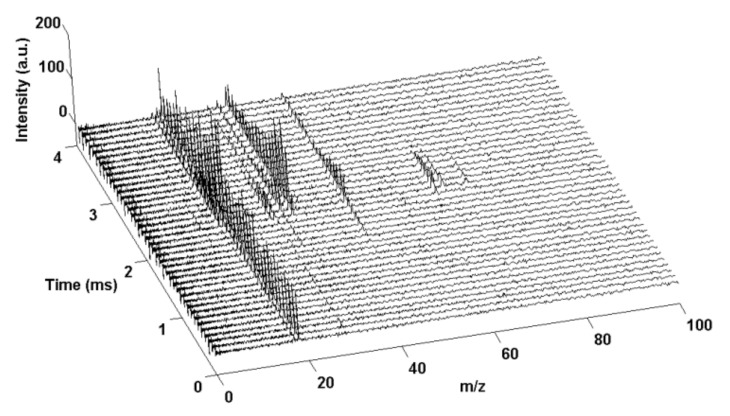
TOFMS time-resolved mass spectra obtained for Al/CuO nanothermite. Reprinted with permission from [164]. Copyright 2010 American Chemical Society.

The system consists of a TOF chamber, an electron gun, and a T-jump segment. A high current DC power supply polarizes both the repulsion and extraction plates. Ionization occurs for a preset time between the plates, after which the voltage on the extraction plate is changed to create a field for ion extraction. The extracted ions flow through a TOF tube and are counted in a microchannel plate (mcp) detector. The process is then repeated according to a preset sampling rate. A T-jump is synchronized with the TOF measurement system to correlate the instantaneous temperature with the corresponding mass spectrum. An example of the obtained time-resolved mass spectra is shown in Figure 9.

This technique found application in examination of nanothermites such as: Al/CuO [164,166], Al/Cu_2_O [165,166], Al/AgIO_3_ [108,115],Al with persulfate salts [106], Al/AgFeO_2_ [167], Al/Ag_2_O [168], Ti/KClO_4_ [116], Al/Fe_2_O_3_ [164], Al/ZnO [164,165,169], Al/Bi_2_O_3_ [169,170], Ti/I_2_O_5_ [117], Si/I_2_O_5_ [117], Al/I_2_O_5_ [117,169] and ternary (based on Al, Si, Ti and I_2_O_5_) systems [117].

#### 3.2.3. Temperature of Reaction

**Figure 10:**
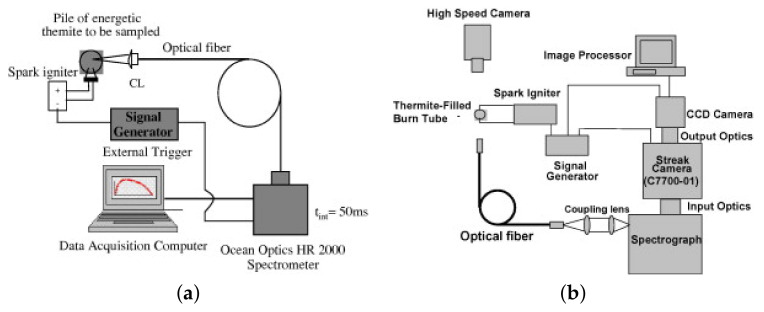
Schematic of time integrated emission collection experiment using an unconfined pile of nano-thermite (**a**) and schematic of temporally resolved spectra and high speed camera data acquisition experiment using a thermite filled burn tube (**b**). Reprinted from [192] with permission of Elsevier.

In [192] the authors presented a methodology for measuring reaction temperatures based on multi-wavelength pyrometry (Figure 10).

In the system shown in Figure 10a, a thermite weight of about 10 mg was placed on a spark igniter. Once initiated, the generated spectrum was recorded using a spectrometer and data acquisition system. In addition to determining the burning temperature, the authors also determined the AlO content from these spectra. Determination of the AlO content allows postulation of the gaseousness of the tested composition. In the second test system, the nanothermite under test was placed in an acrylic tube 89 mm long and 6.35 × 1.59 mm in diameter. One end of the tube was closed with an ignition device. The combustion temperature was measured at a distance of 50 mm from the initiation point. The generated radiation spectrum through an optical fiber and a set of collimating lenses went to a spectrometer and further to a streak camera (measuring changes in radiation intensity as a function of time). The radiation spectrum was recorded by a CCD camera and processed by an appropriate processor. The combustion velocity was recorded with a high-speed camera placed transverse to the pipe axis. In both cases, the reaction temperature was determined based on Planck’s equation [193]. Similar test systems are discussed in the works [194,195]. In [113], to estimate the burning temperature, the authors used color factor pyrometry (taking into account the ratio of three base colors) based only on a high speed camera. The camera was calibrated based on Planck’s law and the assumption of a perfectly gray body using an infrared source.

**Figure 11:**
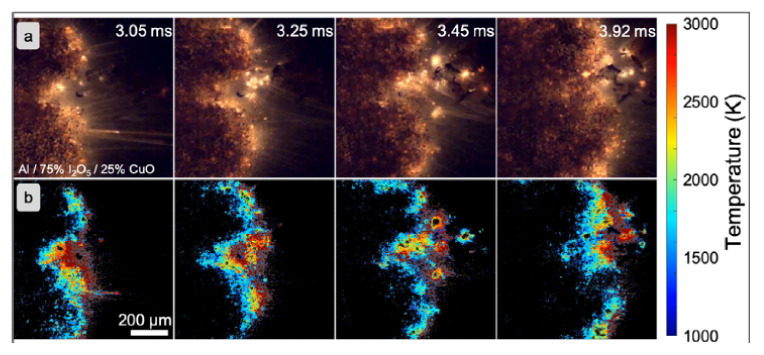
High-speed microscopy images for the Al/I_2_O_5_: (**a**) high-speed microscopy images; (**b**) corresponding temperature maps as calculated by color ratio pyrometry. Reprinted from [196] with permission of Elsevier.

In paper [196], the authors performed a series of studies on 3D printed nanothermite compositions. After printing on the glass tray, the samples were mounted on a translation table and ignited. Propagation of the reaction front were recorded using a high-speed camera coupled with a long-range microscope calibrated for color pyrometry (Figure 11). A detailed description of the idea, is presented in [197]. Example images captured by the authors with up mentioned technique are presented in the figure below.

**Figure 12:**
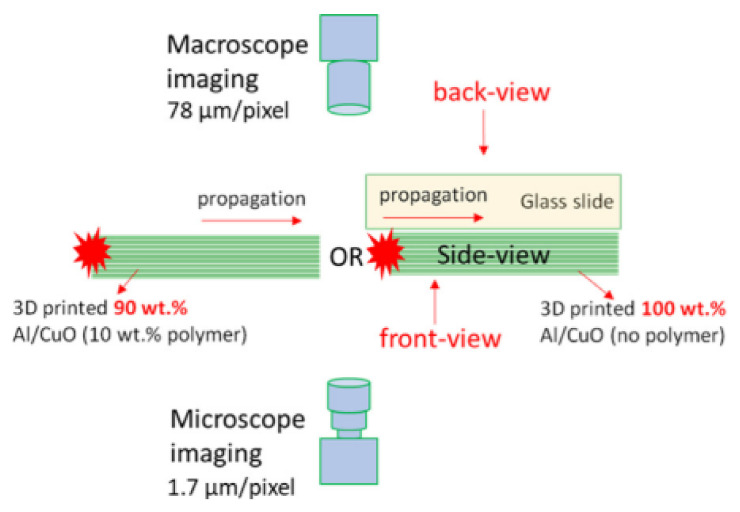
Schematic of the experimental setup. Reprinted from [198] with permission of Elsevier.

A similar setup (Figure 12) was used by authors of [198]. Main difference is form of the testes explosive. The scheme of the setup is presented below.

Described in this chapter techniques, have been used in examination of nanothermites such as: Al/CuO [172,192,197,198,199,200], Al/Bi_2_O_3_ [172], Al/MoO_3_ [192,197,199,201], Al/Fe_2_O_3_ [197,199], Al/WO_3_/Fe_2_O_3_ [197] or Al/I_2_O_5_ [196].

### 3.3. Velocity of Combustion

One of the most important high-energy parameters that characterize nanothermite and NSTEX compositions is the burning velocity or detonation velocity. The characteristic feature of nanothermites is a dramatically high burning velocity, close to the values already characteristic of the detonation process. However, it should be remembered that burning of nanothermites does not follow the detonation mechanism [202]. Usually tests are performed in closed vessels, such as tubes. As it is known, among the key parameters limiting the burning rate, the following should be mentioned: composition, mechanism, thermal effect and reaction kinetics, charge diameter, applied cover and its parameters, particles size of raw materials, degree of homogenization, density, initiation method. The paper [124] discusses the construction of a system to study the burning rate of nanothermite in microchannels. The test system consists of a steel body connected to the lid with screws, with a hole milled in it allowing for mounting the kit containing the nanothermite and recording the burning processes with a high-speed camera. The construction of the cover allows us to initiate the tested nanothermite using a spark igniter. A steel plate with a milled channel containing the tested nanothermite, sandwiched between PMMA plates. Such a “sandwich” is placed in the slot of the steel base.

In [92], the tested nanothermite, was placed between steel plates of size 10 × 20 mm. The layer thickness of the test material was 0.1 mm. The system was thermally initiated using the ignition composition. The recording of the burning rate in this system was solved by using ionization pins, placed at a known distance and calculating the burning rate based on the recording time.

In many works, the burning velocity test is performed for channels milled out into the support. The key difference from previously described methods is related to the use of open channels. Higher burning rates are achieved and the process stabilizes faster when a closed geometry of channel is used to reduce reaction product spreading and heat loss.

In [203], a different type of system for measuring the burning rate was described. It consisted of a silicon matrix in which nine 1200 × 300 × 100 μm channels were fabricated by deep reactive ion etching and terminated with a 500 μm diameter cylinder at the ignition point. The studied systems (including Al/CuO and Ti/KClO_4_ compositions) were initiated with a solid-state bridge. The measurement of the burning rate was carried out using a camera. Similar systems were described in the works [204,205]. In the paper [206] the Al based nanothermites, with Fe_2_O_3_, Fe_3_O_4_,CuO and Co_3_O_4_ as the oxidizers, was examinated by using 1 × 1 × 30 mm channels hollowed in polycarbonate, into which 25 mg of the nanothermite under study was elaborated each time. In [130], a 130 × 5 × 5 mm channel was used to study Al/iodate salts nanothermites.

In some articles, the authors burn tested materials in form of trays or prepressed or printed cylinders and evalute the burning phenomena in open air conditions. In work [25], the authors examined the combustion velocity of nanothermite fibers produced by electrospinning. In [207], the authors performed the combustion test of 3D printed grandient Al/PTFE structures with different mass ratios and diameters. The velocity was measured with high speed camera. In work [208], the authors burn in mentioned conditions 3D-printed nanothermite filaments, and based on fast camera records, the FPV, time of ignition, time of unsteady burning was determined. In [115,209], combustion tests was performed on loose packed trails on the aluminium support to study the combustion velocity. In work [115], the authors performed a combustion test, by ignition of trail of nanothermite in the open air and recorded the process with high speed camera.For these conditions, authors calculated FPV, but they did not reported data about dimension of trail and density of material. The conditions during that test was extremely different than in previously reported tests, and can not be comparison. In work [210] propagation velocity of Al based nanothermites with CuO, WO_3_, MoO_3_ and Bi_2_O_3_ were measured for small bulk density samples placed in a groove made in a metal die, igniting the sample from one end. The propagation velocity was determined using photodetectors. The same setup was used in [211] to study of Al based nanothermites with CuO, Fe_2_O_3_,WO_3_, MoO_3_ and TiO_2_ [212] and Al/MoO_3_.

**Figure 13:**
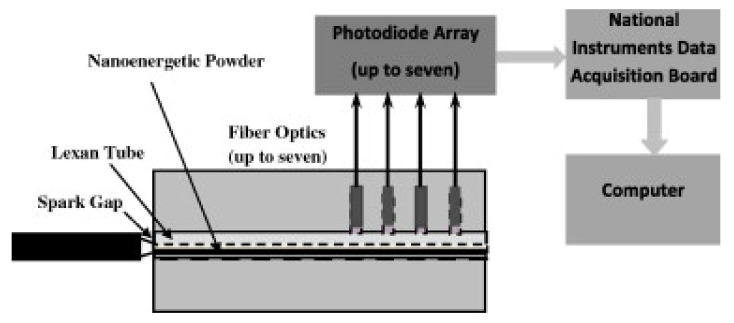
Schematic of the experimental setup for combustion wave speed measurement. Reprinted from [183] with permission of Elsevier.

In a considerable number of works concerning this issue, the burning velocity test is carried out for compositions elaborated into tubes of various geometries, usually made of glass, PMMA or PC. The recording of the burning process, usually with the use of high speed cameras, allows us to collect a large amount of measurement data and to accurately describe the development of the ignition process, the stabilization of stable burning and to determine the magnitude of fluctuations during burning. Conducting the study in this way allows simultaneous acquisition of other data as well. In the work [124], Al/MoO_3_ nanothermite was studied for diameters of 0.48, 1.01, 1.85 (glass tube) and 3.63 mm (plastic tube). In [60], the combustion of Al/CuO nanothermite in the form of compressed pellets was compared with that of thermite sandwiched in a tube. In [92] the Al/CuO was studied in tubes with internal diameter equal to 2.4 mm, but using ionization pins spaced at known distances for FPV detection, instead of typically used high speed camera [127]. In [9] the RDX based NSTEX’s and id [183] the RDX, AN or Cl-20 based NSTEX’s were studied in plastic tubes with high speed camera (Figure 13).

In the work [183], the system shown in the figure above was used. A plastic tube, 3.2 mm in internal diameter and 101.6 mm in length, was elaborated with 200 mg of the composition under study. The element thus prepared, after being closed in a steel die, was ignited with a spark igniter. The burning velocity was calculated on the basis of the operation time of the corresponding photodetectors. The same measurement system was used to study Al/CuO nanothermite [175] in parallel with the “on chip” measurement, in [185] to study Al/CuO, in [100] for Si/NaClO_4_ [180], for Al/Fe_2_O_3_. In [16], a system similar to the above has been described, where nanothermite placed in a plastic tube is enclosed in a steel matrix equipped with both photodetectors and pressure sensors. Such an arrangement allows for simultaneous detection of the FPV as well as the speed and position of the pressure wave. A further part of the system, similar in design to the shock tube, allows for the measurement of pressure characteristics.

**Figure 14:**
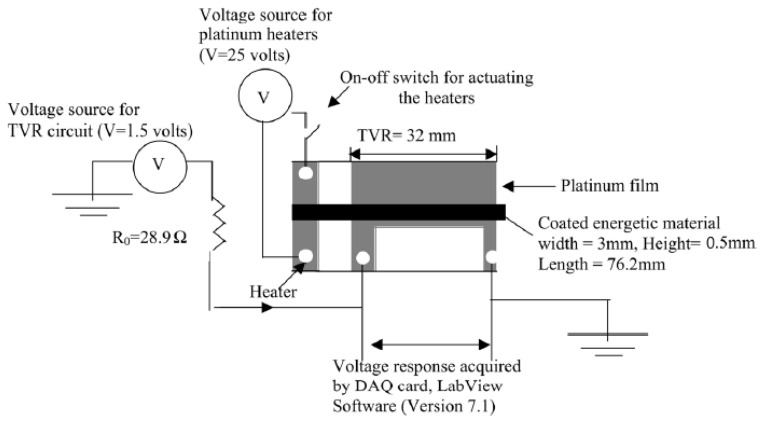
Schematic of voltage divider circuit used for on-chip burn rate measurement. Reprinted from [215] with permission of Taylor & Francis.

A continuous velocity probes, in designs similar to described in works [213,214] usually are not use in nanothermites study. Because of their diameters, typically in the order of tens of mm they are too big to use in charges with really small diameters. This kinds of probes also require very precise assembly in the charge axis. Probes of mentioned above sizes are going to absorb large amount of heat, so they will strongly affect the burning process. The rule of work, of continuous probes relies on change of resistance of conductor, during the welding of them by the shock front. In work [215], the authors decribed a continuous probe velocity meter system (Figure 14), based on a thin platinium layer. The authors deposited the layer of Pt on glass slides covered with alumina shadow mask. After removing of the alumina layer and connecting the wires authors deposited the nanothermite by applying multiple layers of slurry one over another. The scheme of whole test system is presented in the figure below.

Once the sample was initiated, the propagation of the burning front caused a change in the resistance of the TVR film, which was recorded as a voltage change when the system was supplied with DC current. The problem of delay of the system response, the authors solved by starting the data acquisition before the sample was ignited (approximately 1 s before ignition). A similar setup was already described, however [160].

### 3.4. Open Air Combustions

In many studies, the combustion test of uncovered charges have been examined to compare tested nanothermites in the same ignition setups.The influence of the basic composition, additives, particle size, preparation technique or initiation mechanism can be compared and characterized using a high speed camera coupled to the sample initiation system. This test allows the determination of ignition induction time, burning time and with the appropriate instrumentation, also the burning temperature or burning intensity. Usually, the tested nanothermite is placed on the ground, ignited and the burning process is registered by a high-speed camera. For example in work [17], the authors ignite tested compositions (Al/CuSO_4_·H_2_O) by hot-wire method. In [24] this method was used to evalute Al/CuO/Cl-20 NSTEX, in [119] for Al/WO_3_, Al/CuO/GO [159]. The authors of [158] used similar method but with different ignition mechanism was used to study Al/CuO/SWCNTs. Some variants of mentioned test were made in [29] to study Al/NiFe_2_O_4_,^74^] to study Al/MoO_3_ or [144]—Al/PFAA. In [207], the authors performed the combustion test of 3D-printed column structure, when the combustion stage lead to deflagration. In work [216], the authors performed similar tests for the Al/RDX/PVDF composition.

It should be noted that although different variants of techniques involving the open burning of tested materials allow the measurement of a number of parameters (e.g., temperature of reaction, optical emission, ignition delay), they do not allow direct comparison of results between researchers. Different methods of ignition, weights of tested compositions, their geometry or methods of application are used. In many papers the authors do not specify in a comprehensive way the parameters that would allow referring to the test described by them and its replication by another team.

The primary issue in this aspect is that of the large variation of conditions, in which measurements are carried out. Due to this fact, it is immeasurably difficult to accurately compare the results of many recent works, as even a difference in sample size can significantly affect the achieved results. to exemplify, conducting combustion in small, milligram-level samples, which can enter into secondary reactions with the air available in the open air, exposed to huge heat losses is vastly different than the combustion of larger samples and is in no way comparable to the conditions, in which such materials are typically utilised. In summary, these techniques are appropriate to compare different nanothermites or NSTEXs, but only in exactly the same study setup.

### 3.5. Pressure Parameters

Together with the burning velocity, pressure parameters are the most important data characterizing the high-energy materials. The pressurization rate can be used as a measure of reactivity in nanothermites since it has been found the correlation between them. Pressure of high-energetic process and external pressure strongly affect on the combustion mode of non-detonating explosives, such as pyrotechnic compositions. Particular attention should be paid to the pressure bomb test. This technique allows us to estimate character of combustion reactions, value the gas volume per mass of composition and gives many data to determine the mechanism of reaction; maximum pressure, pressurisation rate, time to reach maximum pressure, time of overpressure and impulse. Very often the pressure bomb experiments can be considered as adiabatic and the pressure increase can be related to the energy produced in combustion. Typically pressure bomb is a closed, thick walled vessel with constant volume, stocked at least in pressure sensors and ignition system. Usually, pressure sensor is placed transversely to the sample and the volume of the chamber is several times larger than the volume of the sample. This simple kind of pressure bomb was used, e.g., for examination of Al/Fe_2_O_3_ [104,208], Al/CuO/Viton A [93], Al/Fe_2_O_3_/RDX [8], Al/CuO/CB [217], Al/CuO/SWCNT [158], Si/KClO_4_ [100], Al/CuO [208], Al/PTFE [208], Al/I_2_O_5_ [177], Al/CuSO_4_·5H_2_O, Al/Bi_2_O_3_ [177,208], Al/FP [11], Al/ECPs [218] or Al/CuO/NC/Cl-20 NSTEX composition [24].

**Figure 15:**
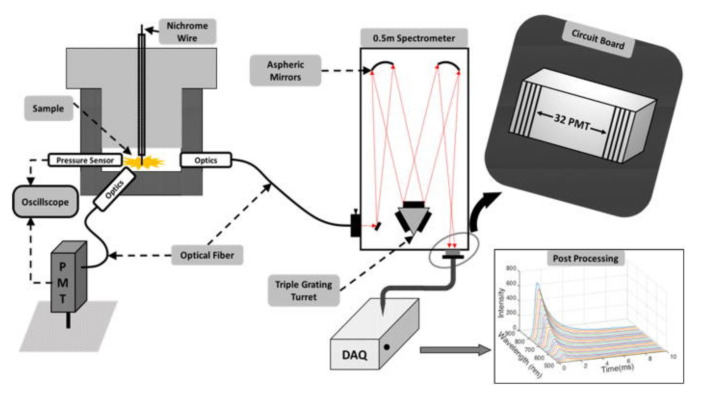
Schematic of the experiment consisting of the pressure cell and attached diagnostics. Reprinted from [197] with permission of AIP Publishing.

Very often, pressure test is connected with registration of emission intensity or spectra analysis. In work [197], the authors described the setup, for simultaneous registration of pressure, emission and temperature of reaction. The course of pressure as a function of time is directly related to the velocity of the flame [115], and thus to the reaction rate. In previous work, a strong relationship between gaseous product release, pressure peak, and pressurization rate has also been noted. These relationships, however, are not always correlated with optical emission intensity and reaction temperature [197]. Simultaneous measurement (Figure 15) of these correlations rapidly increases the possibility of studying the mechanisms of combustion reactions.

The pressure sensor, fiber optic coupled to a photomultiplier tube, and fiber optic coupled to a spectrometer were used in the pressure bomb discussed here. Ignition of the sample was initiated using the hot-wire method. The use of such a system allows data describing the pressure characteristics, reaction temperature changes, and optical emission of the burning process to be linked. Similar setups were used in works [117,188]. The system in a simpler variant, limited to a single measurement of emission and pressure, was used, among others for examination nanothermites as Al with iodine oxides or iodine acids [118], Al/Ag_2_O [168], Al with Bi(IO_3_)_3_, Cu(IO_3_)_2_ and Fe(IO_3_)_3_ [219], Al/I_2_O_5_ [169], Al with NaIO_4_ and KIO_4_ [105], Al with CuO, Fe_2_O_3_, Bi_2_O_3_ [220], Al with CuO, Cu_2_O [166,221], Al/AgIO_3_ [115] and Al/WO_3_ [179].

**Figure 16:**
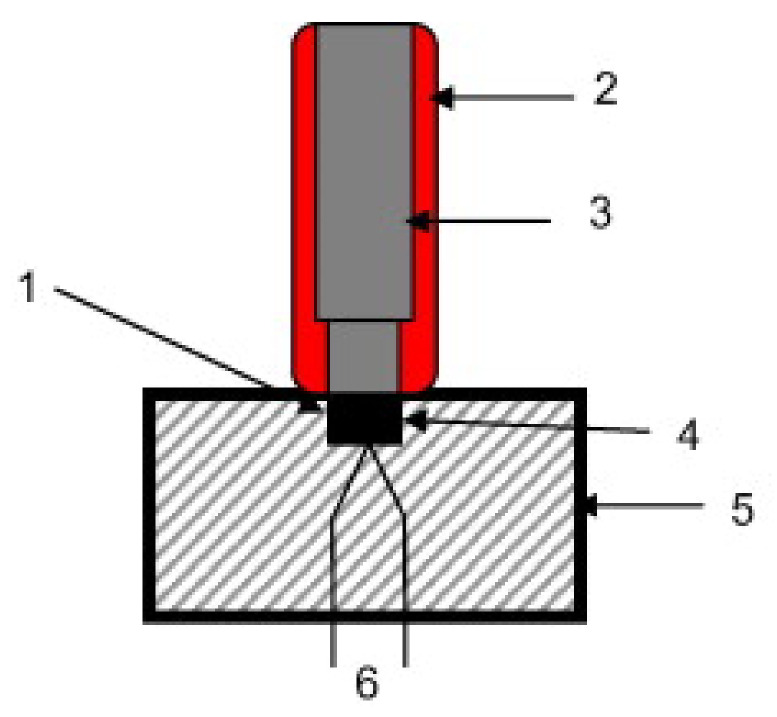
Schematic of the experimental setup for pressure–time measurements (1) combustion chamber, (2) pressure sensor holder, (3) pressure sensor, (4) test material. (5) metal support, (6) hot wire. Reprinted from [183] with permission of Elsevier.

In [180,183,222], the authors desisgned the different kind of pressure bomb (Figure 16).

In a cavity made in a steel block of specified dimensions, the authors placed a nanothermite sample of known mass so as to fill the entire available space. The authors used a chromonickel wire as the initiation system, directly contacting the nanothermite under test. A pressure sensor was mounted above the sample of nanothermite under test, thus closing the chamber. The described method of dispensing and mounting allowed to ensure reproducible density of the compositions under study. However, depending on the authors, different chamber volumes of 0.6–25 cm^3^ and different weights (up to 50 mg) are used. The authors of this paper [183], using a chamber volume of 0.06 cm^3^, indicate that convective effects are nullified by using such a small chamber, fully fill the chamber under study, and facilitate the analysis of NSTEX compositions. In addition, the authors of the aforementioned work performed a number of tests for chambers of different volumes (0.06–16.5 cm^3^) keeping the nanothermite mass constant. Such study allowed to determine the amount of energy released during the reaction. Note also the limitation of secondary afterburning reactions of nanothermite occurring with oxygen available in the chamber volume. Similar in construction, pressure bombs was described in works [146,185,223].

In work [224], the authors described the setup of drop-hammer impact apparatus, connected with the pressure sensor, based on the [225]. The nanothermite composition was pressed into the form of cylinder with density equal to 90% of TMD. After that, pellet was placed inside the steel body, between pressure sensor and the firing pin. The body was placed under a guide rail on which the hammer was lowered. The hammer, with adjustable mass and drop height, initiated ignition by striking a pin, which impact the sample.

In work [226], the authors used a Crawford bomb for examination of external pressure impact on burning parameters of Si and Al based nanothermites.

**Figure 17:**
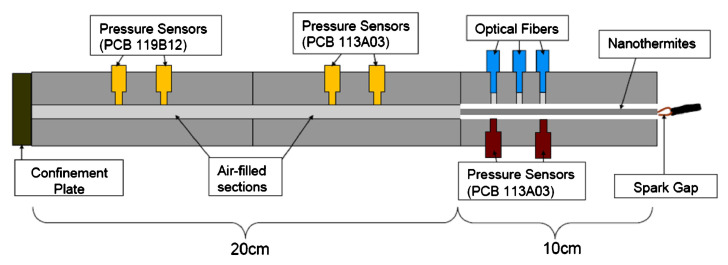
Schematic of the shock-tube setup used for the measurements. Reprinted from [16] with permission of AIP Publishing.

A fascinating shock-tube setup (Figure 17), allowing the simultaneous registration of pressure and burning velocity in function of a distance, were described in work [16]. The main advantage of this technique, is a possibility of appointment of correlation between FPV and pressure wave velocity, which is strongly connected with initiation and burning mechanism.

In the system presented, nanothermite elaborated into a plastic tube was placed in a one-sided closed channel of a mounting block, equipped with pressure sensors and photodetectors. The arrangement of pressure sensors, allowed simultaneous acquisition of pressures in the space filled with thermite and in the shock tube. In work [227] the tested nanothermite (Al/MoO_3_) was placed in an acrylic tube, which was placed in an acrylic block equipped with a set of 6 photodetectors and 6 pressure sensors spaced every 1 cm. Unlike the system described earlier, no closure of the tube was used on the side opposite the ignition. The entire system was mounted in a steel body equipped with a window allowing recording of the burning process using a high-speed camera. The same or very similar setup was used to examine the Al/CuO and Al/MoO_3_ [199], Al/Viton/Teflon and Si/Viton/Teflon [226], Si based nanothermites [125], Al with CuO, MoO_3_, Bi_2_O_3_ and WO_3_ [210], Al/MoO_3_ an Al/CuO [199].

In [161], the authors measured the combustion velocity of Al/HTPB composition in quasi-static pressure conditions. The tested material, after being cut to the given dimensions and side wall inhibition, was burned in a laboratory combustion chamber, in a nitrogen flow adjusted in real time to ensure a constant pressure value.

### 3.6. DDT-Tube

**Figure 18:**
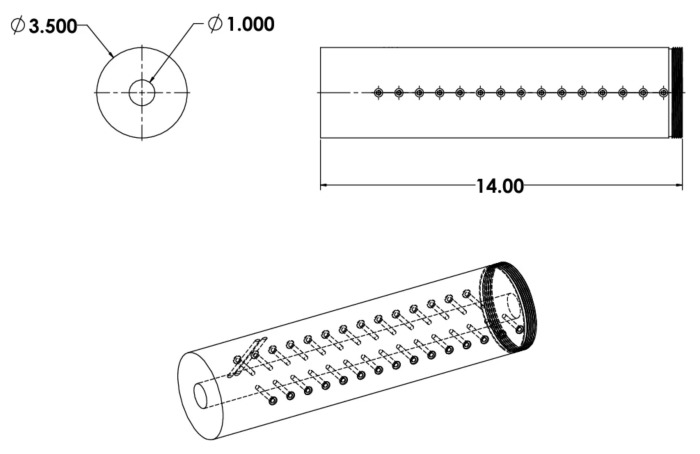
Schematic depiction of a DDT Tube; unit: inches. Reprinted from [229] with permission of Springer Nature.

A DDT-Tube type test system is an interesting set-up, consisting of a thick-walled, usually steel tube, with a high L/D ratio, provided with a massive closure of one of the outlets [228]. The other outlet is closed by an ignition system of various types. Various types of probes (short-circuit, ionisation, photo) or sensors are placed transverse to the pipe axis; additionally, it is possible to enrich the system with continuous type probes, recording of X-ray images, recording of inert spacers position. A DDT-Tube type system can be depicted as follows (Figure 18).

Measurement with this technique allows us to accurately characterize the DDT process; to determine the possibility of the process occurrence, the DDT transition time, the detonation development distance, determine the type of DDT process [230], to accurately characterize the propagation mechanism of the high-energy transition and indirectly to evaluate the performance. In work [8], RDX/Al/Fe_2_O_3_ composition was investigated by DDT-Tube method. In work [183] by using a tube made of polycarbonate, the authors determined the detonation capacity and detonation velocity of compositions based on a mixture of Al/CuO nanothermite with NH_4_NO_4_, Cl-20, and RDX. In [9], the authors investigated the development of the detonation process in a mixture of RDX with Al/WO_3_ and Al/Bi_2_(SO_4_)_3_ nanothermite and the ability of these NSTEX-type compositions to initiate a secondary PETN charge. In [231], the authors performed the DDT study on the ammonium dinitramide based NSTEX compositions. Tests were performed in PMMA tubes, with 3 mm internal diameter and 150 mm long. Tests in similar setup, were performed for the TiH_2_/KClO_4_ composition in [232].

Interesting from the point of view of high-energy transformations of NSTEX compositions is rather the DDT type II mechanism. According to works [230,233], this mechanism is characteristic for solid explosives with low densities and high porosity and for aerosols. Occurrence of DDT process in the form of type I or II depends on such parameters as: type of explosive, burning velocity in the bare state, fragmentation and proportion of individual fractions, morphology of particles, configuration of charge—envelope material, its dimensions and method of initiation [230,233]. The described above method, allows us to determine the parameters and type of DDT process. However, the disadvantage of this technique is the necessity to provide a large mass of high-energy material, which may be related to its low popularity in the analysis of NSTEX-type compositions.It should be noted that most of the tests described above were conducted in plastic pipes instead of steel pipes. This makes it possible to observe the DDT process using an regular fast camera, while at the same time mitigating the conditions for the development of the process by not providing sufficient holding of the high-energetic decomposition products.

### 3.7. Thrust Measurement

Among the potencial application of nanothermite and NSTEX, we can highlight small rocket motors and propulsion systems, e.g.,microthrusters or executive control systems for aerospace technologies. In works [234,235], the authors described a system for measurement of thrust with or without convergent–divergent nozzle, presented in the Figure below.

In this system, the force sensor placed inside of support plate was connected vertically with thrust motor, the burning process was observed with high speed camera. As an ignition system, authors used hot-wire method. The authors examine Al/CuO nanothermite in the function of density (even up to 60% TMD), length of the burning chamber and nozzle design. In [62], the authors used similar setup to examination of the Al/KClO_4_/NC/GO nanothermite. The burning chamber was made from acrylic tube, and was supplied in convergent-divergent or cylindrical nozzle. The set of measured devices include fast speed camera, force transducer and set of photodiodes with filters. The burning chamber with nozzle was placed inside external chamber, which implies possibility of carry out tests in different atmospheres and pressures. The samples was ignition by laser radiation. Used setup allowed authors to measure the AlO signal, which can be used to calculate the temperature of the reaction and give information about mechanism. In work [235], the authors described system for measurement of thrust for MEMS-based nanothermite thrusters. In [236], the authors used similiar to [234] system. Except for that, authors described also a fascinating system for measurement of High-G tolerance of nanothermite thrusters.

In [237], the nanothermite chamber was closed with a plane, placed at an angle of 45 degrees, redirecting the gas stream to a cylindrical nozzle placed transversely to the chamber axis. Except for the statistic tests, authors discussed the setup for evaluate thruster performance in a kinetic system. In this arrangement, the thruster was threaded into a fixed axis rotating arm mounted on a vertical stand with the aid of bearings. The ignition of the tested thruster caused the arm to move in the horizontal plane, the movement of which was recorded using a frame camera. The use of such a system made it possible to calculate the efficiency of conversion of chemical energy into mechanical energy, kinetic energy of the system, drag work and friction force. The chemical energy was determined on the basis of a calorimetric bomb test.

In [238], the authors presented the 3D-printed micro-thrusters, elaborated with Al/Cu(IO_3_)_2_ nanothermite. The examination of prepared thrusters included dynamic and motion of the rocket test. The body of the rocket after elaboration was placed in horizontal axis and ignited. The motion of the rocket was registered with high speed camera. By analysis of the pictures authors evaluted the parameters specific for the thrusters (thrust force, impulse and specific impulse).

The potential application of nanothermite or NSTEX composition in, e.g., microthruster, causes that it is necessary to determine the thrust of a given system both in static and dynamic conditions. Even compositions with low pressure parameters or low combustion velocity, can provide excellent thrust parameters, appropriate for limitation of given system (e.g., time of work, total thrust, impulse). The described technique is rather designed for the examination of finished systems, than characterization of compositions.

### 3.8. Ballistic Mortar

The method of determining the work capacity using the ballistic pendulum method, is based on the detonation of the test charge, with a specified density and geometry in the pendulum arm socket. Based on the pendulum swing and the pendulum swing values for the reference charges, the relative work capability [239,240] is determined. This technique also allows the estimation of explosion strength based on appropriate equations.This method is still relevant due to the huge amount of data published for different high-energy materials and the simplicity of execution. In the work [241], the authors performed a study for compositions containing TNT with Al, Al/CuO nanothermite or Al/CuO/HECNTs nanothermite. In paper [154] the study was performed for TNT with Al or Al/MnO_2_ nanothermite addition.

The main disadvantage of the described method, is is the need to provide a significant mass of the explosive (approximately 10 g for each test) and the fact, that this method is primarily a comparative method that does not provide a large amount of data to characterize the sample. Methods such as underwater text explosion or cylindric test could provide more data, including data related with high-energetic decomposition process parameters such as JWL equation [242,243].

### 3.9. Brisance

The brisance of a high-energy material is a parameter that is connected mostly with the detonation pressure. One of the techniques to investigate this parameter is the Hess test and the Kast test. In the case of both tests, the idea is to measure the crush of a cylinder (respectively made of lead and copper) after detonation of a high-energy material of a specific density and geometry. The obtained value is referred to a standard high-energy material or after reference to standard tables, determines the value of overpressure at the front. In the paper [244], the authors realized the Kast test for TNT-based compositions with the addition of nano-Al, Al/CuO nanothermite or Al/Fe_2_O_3_.

Similar to the ballistic mortar tests, the main disadvantage of the mentioned above methods is fact, they are just reference methods. Except for that, in Kast test there is need to use a large quantities of lead, which is toxic for humans and environment. The determination of brisance in both tests, is based on the assumption that the cylinder compression is proportional to the brisance of an explosive charge, or momentum given by explosives respectivetly. In both test, the cylinder material, hardens under load which changes its strength parameters. It lead to situation, where the energy required to crush subsequent sections of the element, grows up. The comparing of strong explosives with these methods are limited by significant errors. Probably, better techniques include the measurement of detonation pressure techniques, e.g., water test [245], manganin gauges tests [246] or electromagnetic gauge test [33]. This approach is possible as there is a strong correlation between brisance and pressure at CJ point [33].

## 4. Applications of Nanothermites and NSTEX

Thermite formulations are not high-explosives, even though their energy density is higher than that of typical high explosives [18]. Their high energy density is utilised so as to rapidly achieve high temperatures for the purpose of, e.g., cutting metal or welding metal elements together, without the need for utilising specialised or heavy equipment, as seen in the case of various types of electrical welding. Due to the fact that welding with thermite formulations fills the internal contact surfaces with metal, the produced welds are highly durable, corrosion-resistant and conduct electricity well. The Goldshmidt (aluminothermic) reaction and other reactions jointly labelled as “thermite processes” have also found application in metallurgy, as means for extracting pure metals from their ores without the use of carbon-bearing additives. An example of such a process was the method of obtaining significant amounts of pure uranium developed for the Manhattan Project. Used as combat agents, thermite formulations are classified as incendiary pyrotechnic compositions, and can be used as handheld cartridges or as magnetic grenades. Used when it is necessary to quickly destroy items and other equipment, when there is a high risk of their sudden capture by enemy forces.

In the further part of the preview, other unusual uses of nanothermites, described in the latest literature, will be presented.

### 4.1. Devices Using Nanothermites and NSTEX

**Figure 19:**
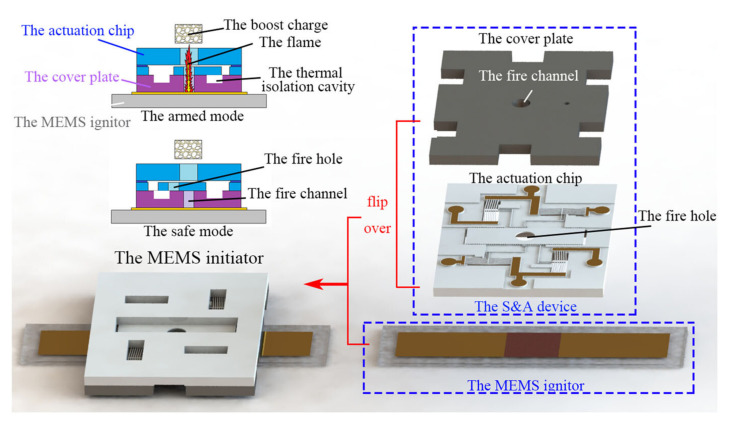
Schematic depiction of the structure of the MEMS initiator. Reprinted from [247] with permission of AIP Publishing.

Nanothermites are particularly well suited for microdevice applications. The high level of tuning and excellent reaction performance in microscale systems make nanothermites a potential solution for a variety of applications requiring exceptional energy properties in both micro and macroscale. Hu [247] in his work proposed novel MEMS initiator with a built in safety-and-arming (SaA) mechanism (Figure 19). The device presented at figure below is composed of the MEMS igniter and MEMS SaA device. The laminated Al/CuO structure is deposited on the MEMS igniter to enhance its energy output. The corresponding SaA device is set on the explosive train. Stimulated by specific electrical signals, the fire channel of the MEMS SaA device can be opened (armed mode) or closed (safe mode). The authors show that the output displacement of the MEMS SaA device is 1 mm, which is enough to block or open the fire channel (ϕ 600 μm). Under the armed mode, 64 V pulsed voltage can trigger the explosion, and the flame height can reach nearly 8 mm. Under the safe mode, the fire cannot come out from the fire hole even when the igniter is mistriggered.

A micro-chip initiator with controlled combustion reactivity is reported [248] and utilises concepts usually applied to microelectromechanical systems (MEMS). The nanothermite composites fabricated in this study consisted of aluminium nanoparticles as the fuel and copper oxide nanoparticles as the oxidizer accumulated on a silicon oxide substrate with a serpentine-shaped gold (Au) electrode. The micro-chip initiator rapidly ignited and exploded when minimal current was supplied. The controllability of combustion reactivity of micro-chip initiator can be made for general nanothermite compositions constituted by Al and various metal oxides (e.g., Fe_2_O_3_, CuO, KMnO_4_, etc). The micro-chip initiator fabricated in this study was reliable, compact, and proved to be a versatile platform, exhibiting controlled combustion reactivity and fast response time, which could be used for various civilian and military thermal engineering applications, such as in initiators and propulsion, welding, and ordinance systems Micro-initiators filled with bismuth and aluminium oxide showed controlled initiation with 100 % efficiency in the firing energy range from 30 to 80 μJ and response times below 2 µs for high power input cases. The microinitiator platforms presented in publication are fundamental enough to be used in a wide spectrum of MEMS pyrotechnic applications. The fast response time is ideal for use in miniaturized, digitized bow thrusters capable of quick correction of missile or microsatellites trajectory. This large production of gaseous products from these initiators is of interest to large-deflection flexible piston actuators for controlling microflow valves or microballoons. Finally, the low ignition sensitivity of the nanothermite found in micro-initiators can be incorporated into secondary explosives or trains firing propellant [249].

A very avant-garde method of generating electricity through a combined heat and power plant is reported [250]. The system and method include a fuel preheat chamber configured to receive nano-thermal fuel, an induction assembly configured to inductively heat the fuel in the fuel preheat chamber, and an electrical power generation subsystem configured to convert heat from the heated nano-thermal fuel to Electricity.

Niollet [251] in his work presented an alternative to traditional pyrotechnic switches with a device based on nanothermite, which is a compact switch, ideally suited to protect against over-current, external disturbances and short circuits of a wide range of devices and systems. The new miniature switch would be miniaturized by integrating a few mg of nanothermites by additive manufacturing methods directly into electronic circuits. The concept is simple and adaptable to many applications: two printed circuit boards (PCBs) are connected to each other to form a 38 mm^3^ airtight cavity. The lower printed circuit board contains the electronic circuits and an ignition element for triggering the switching. The second PCB supports the copper connection as part of the circuit that needs to be disconnected. Once ignited, the nanothermite generates a high-pressure gas pulse sufficient to safely terminate the circuit’s electrical connections in less than 2 ms, well before a short circuit occurs that could lead to an uncontrolled operation such as an accident or disaster.

Lopez [252] in his work presented a WIMP (Weakly Interacting Massive Particle) detector, based on a nanothermite composition. The principle of operation is based on the interaction of WIMP with metal nuclei. WIMP can transfer enough energy to a metal atom to initiate its ignition, which then develops into the entire composition. The system operating in the atmosphere of liquid nitrogen shows a sensitivity of 0.5 eV.

In patent [253] the bridgeless electric explosive device was obtained by modifying the surface of the oxidized carbon fibers on which the nanothermite was deposited. Functional carbon fibers are conductive and heat-conducting carriers, local discharges and heat accumulation are created in nano-sized energy-containing materials, thus obtaining the ignition or detonation functions. The authors point out that in this way the ordering of all components in the nanothermite can be improved to a large extent, the agglomeration between the particles can be reduced, and the reaction rate and energy of the nanothermite can be improved. Functionalization carbon fibers reduce the electrostatic sensitivity of nanothermite. The nanothermite is applied to a produce bridgeless electric explosive device.

Ni et al. [254] replaced lead styphnate in initiator. Proposed initiator was integrated with Al/MoO_3_ multilayer film and bridge wire by magnetron sputtering. The thickness of Al/ MoO_3_ multilayer was 3 μm with period of 20 nm/30 nm. The firing sensitivities of Al/MoO_3_ multilayer film was almost equal to the lead styphnate. The initiator can fire the lead azide in detonator. In paper t is suggested that Al/MoO_3_ multilayer film can be a substitute for lead styphnate and improve the ignition ability and safety of the ignition system.

Patent [255] describes a device in which nanothermites are used to neutralize improvised explosive devices (IED) and unexploded ordnance (UXO). The proposed solution The invention provides a simple method of neutralizing ammunition through the use of nanothermites and binary termites. In the device, the projectile is loaded with nanothermite and fired into the ammunition. The impact causes the energetic materials to react in such a way that the explosive compound or other material in the IED or UXO is burned in a self-propagation mode without an explosion.

Consequently, the weapons are safely neutralized. In patent application [256], the authors provided the design of a detonator iniciator which includes an explosive and an initiator for igniting the explosive, wherein the initiator includes a thermite composition and a tinder mixture which includes silicon and dibismuth trioxide with Al/CuO addition. The application presents new construction of detonators, e.g., Fire chain: Si/Pb_3_O_4_-nanothermite/resin-nanothermite/resin-nanothermite/PETN-PETN) the construction of a micro-miniature igniter based on a micro-heater and energetic structural material was changed in [257]. Energetic structural material in a micro-miniature igniter termite array (fuel Al or Mg metal oxide CuO, Co_3_O_4_, Fe_2_O_3_, Fe_3_O_4_, MoO_2_) with embedded explosive and has waterproof properties, which improves energy release from the igniter, resistance to moisture and resistance the oxidation fuse is improved, ensuring reliable ignition reliability. The structure of a micro-miniature fuse consists of a micro-heater, a structural thermite matrix, an explosive layer and a waterproof layer; the micro heater includes a bonding area and a heating area; a structural termite matrix is formed in the heating area; the explosive layer is embedded in a structured thermite matrix in situ; and the waterproof layer surrounds the structural arrangement of termites.

### 4.2. Materials Based Nanothermites and NSTEX

In the publication [219], the authors propose a method of detecting biological weapons such as bacillus anthracis with the use of nanothermites. The article describes the synthesis of metal iodate aluminized nanocomposites assembled using the electrospray method, which neutralize Bacillus anthracis spores through a combined thermal and chemical mechanism. In this case, metal iodates (Bi(IO_3_)_3_, Cu(IO_2_)_2_ and Fe(IO_3_)_3_) act as a strong oxidant to nanoalumin, causing a very exothermic and violent reaction, while generating iodine as a long-lived bactericide. The proposed nanocomposites show much better reactivity and sporicidal activity than conventional termites based on metal oxides. Zhou investigated the Al/Na_2_S_2_O_8_ compositions, due to the fact that the system generates a lot of SO_2_ as a result of smoking, the authors suggested potential biocidal applications of nanothermite, although no studies in this area were performer [106]. Thus, iodine pentoxide based nanoenergetic gas generators could possibly be used to neutralize spore forming bacteria and the threat of biological weapons Sulivan in his work [168] tested the Al/Ag_2_O nanothermite, as the system allows for the generation of nano-aerosols, the authors postulated the possibility of creating biocidal coatings, although no studies confirming this application have been conducted. The authors in the paper indicate that he morphology of the final product, indicates that a large amount of the silver may not be surface-exposed, a result which would negatively impact the biocidal activity.

Tappan et al. [258] describes a nanocomposite thermite ink for use in inkjet, screen and gravure printing. Embodiments of this invention do not require separation of the fuel and oxidant constituents prior to applying the ink to the printed substrate. the form of particles in which the particle diameter is less than 10 μm, so that when the formulation is heated and the formulation is reactive, the starting material may be selected from any inert material or, alternatively, an energetic material for which the formulation provides the means for initiation.

Method for preparing a high-combustion-speed micro-pipeline based on nanothermite ink in composition Al/CuO/adhesive solvent is presentd in [CN112626635A] describe. The method for preparing the nano thermite high-combustion-speed micro-pipeline comprises the steps that the prepared Al/CuO/adhesive nano thermite ink is loaded into a needle cylinder, and extruding and directly writing are carried out through a core-shell nozzle to obtain the nanothermite hollow fiber with a certain diameter. The hollow fiber has good self-supporting performance without collapse, a cavity and the fiber wall have good coaxiality, and the combustion performance of the nanothermite is improved by well combining the advantages of the structure of the nanothermite. In [259], the authors presented the concepts of using nanothermite in the copper ferrite/GO/Al system as a catalyst for solid fuel combustion, according to the authors, the proposed nanothermite has a better catalytic effect on the thermal decomposition of energetic materials (such as RDX) compared to the single-component CuFe_2_O_4_, it realizes fast combustion in a steady state solid fuel and reduces the pressure gauge.

Patent [260] describes using thermite reaction methods of synthesis namometer aluminium oxide particle reinforced composite material in a high gravity field. Thermit in a powder form is poured into reaction vessels rotated at a high speed. The thermite is ignited so that all the reaction products in the reaction vessels are in a molten state; and under the action of the high gravity field, metal melts with higher density in the reaction products are located on the inner surfaces of the reaction vessel, and form casting pieces after being cooled, and the melts which have lower density in the reaction products and poor wetting properties with the metal melts are covered on the surfaces of the metal melts, and form molten slag after being cooled.

Quin [261,262] describe an interesting method of using nanothermite in order to obtain a micro-nano particle reinforced aluminium-based composite. By adopting the method, composite reinforcement of the substrate is realized by particles of multiple scales and multiple types, the obtained aluminium-based composite material has the advantages of high intensity, high wear resistance and the like, the bending resistance and Brinell hardness of the aluminium-based composite material are increased by over 50% and 73% compared with those of the conventional aluminium alloy respectively, and the friction coefficient is lowered by over 25%. The patent [263] describes an an innovative method of using nanothermites setting a plug for well bores undergoing plugging and abandonment operations. The methods include placing the locking device below the plug position and creating a void above the locking device; placing a heat resistant base plate over the blocking device and introducing nanothermite clusters and expandable bismuth alloy granules into the void; igniting the thermite and cooling the molten alloy to form a permanent plug in the void.

## 5. Conclusions

Classically defined termites are limited to gasless pyrotechnic compositions, usually based on Al. The development of nanothermites and the nature of their reactions makes it necessary to consider a wide group of compositions based on various components as their nanometric equivalents. The need for a comprehensive study of the properties of this group implies the use of a huge base of research techniques and the creation of new ones dedicated to this group. The number of factors affecting nanothermites in an extremely rapid manner is enormous, which implies the necessity of carrying out a huge number of studies.

However, one should keep in mind the multitude of scientific challenges that must be overcome in order to fully understand, characterize and exploit the potential of this group of high-energy materials. Among them, it is necessary to look into issues related to the mechanism of reaction and ignition, the effect of phlegmatization, aging resistance, the use of perspective fuels different from the most popular one—aluminium, or the question of optimization of composition parameters.

Among the issues requiring special attention is the topic of high sensitivity and aging resistance of nanothermites. Many works do not touch this extremely important issue. Meanwhile, extremely high sensitivity may prove to be an inhibiting factor for nanothermite use. Additions of phlegmatizers, even high-energy ones such as nitrocellulose efficiently influence the burning processes and further—the parameters of whole composition. At the same time, a feasible task is also to increase the safety of explosive devices due to tunable properties of nanothermites.

Another challenge to overcome is the issue of scaling up to industrial scale methods of nanothermite preparation. Much of the currently using methods have high cost, are complicated and require highly skilled operators. The properties of nanometric compounds are strongly dependent on their quality, e.g., an absolutely small difference in fineness can result in several times different burning velocities. Despite these considerations, these methods allow for a completely new and unique approach to the construction and design of blasting agents and devices, allowing to exceed the limits resulting from the properties of typical high energy materials. A major problem standing in the way of the development of this group of high energy materials is the limited knowledge of the toxicity of the components and products of their reactions. This is a particularly important issue in the era of worldwide efforts to reduce the utilization of toxic and harmful compounds, also in the explosives industry.

Properties of nanothermites discussed earlier, placing them in the place of a missing link between classical explosives capable of detonation and pyrotechnic compositions, which may enable their versatile application. The demonstrated applications of both nanothermites and NSTEX compositions described in this work allow to put forward a hypothesis on the possibility of limiting or even giving up completely the use of primary explosives based on lead compounds such as lead azide and TNRO.

## Abbreviations

The following abbreviations are used in this manuscript:

CL-20	2,4,6,8,10,12-Hexanitro-2,4,6,8,10,12-hexaazatetracyclo[5.5.0.0^3, 11^.0^5, 9^]dodecane
NSTEX	NanoStructured Thermites and Explosives
PETN	2,2-Bis[(nitrooxy)methyl]propane-1,3-diyl dinitrate
RDX	1,3,5-Trinitro-1,3,5-triazinane
TMD	Theoretical maximum density
TNT	2,4,6-Trinitromethylbenzene
TNRO	Lead(II) 2,4,6-trinitrobenzene-1,3-bis(olate)
